# Cross-Species RNA-Seq Study Comparing Transcriptomes of Enriched Osteocyte Populations in the Tibia and Skull

**DOI:** 10.3389/fendo.2020.581002

**Published:** 2020-09-24

**Authors:** Ning Wang, Corinne Niger, Nan Li, Gareth O. Richards, Tim M. Skerry

**Affiliations:** ^1^Department of Oncology and Metabolism, Mellanby Centre for Bone Research, University of Sheffield, Sheffield, United Kingdom; ^2^Department of Neuroscience, Sheffield Institute for Translational Neuroscience (SITraN), University of Sheffield, Sheffield, United Kingdom

**Keywords:** osteocytes, RNA-Seq, Wnt signaling, cross-species, bone remodeling

## Abstract

Local site-specific differences between bones in different regions of the skeleton account for their different properties and functions. To identify mechanisms behind these differences, we have performed a cross-species study comparing RNA transcriptomes of cranial and tibial osteocytes, from bones with very different primary functions and physiological responses, collected from the same individual mouse, rat, and rhesus macaque. Bioinformatic analysis was performed to identify 32 genes changed in the same direction between sites and shared across all three species. Several well-established key genes in bone growth and remodeling were upregulated in the tibias of all three species (BMP7, DKK1, FGF1, FRZB, SOST). Many of them associate or crosstalk with the Wnt signaling pathway. These results suggest Wnt signaling-related candidates for different control of regulatory mechanisms in bone homeostasis in the skull and tibia and indicate a different balance between genetically determined structure and feedback mechanisms to strains induced by mechanical loading at the different sites.

## Introduction

Patterning during embryogenesis accounts for the development of all the specialized tissues and organs in multicellular organisms (1). Skeletal patterning accounts for the shape and structure of different bones and their behavior during life. However, mechanical forces provide additional potent influences on most parts of the skeleton, both before (2, 3) and after birth (4). This process of skeletal adaptation tunes bone strength to prevailing functional demands, adding material above a genetically determined baseline structure that forms and is retained even in the absence of loading stimuli.

The physical demands on bones, and therefore their optimal structures vary with their site in the skeleton. These form/function relationships vary widely throughout the skeleton (5), but we can consider examples at the opposite ends of the spectrum. Long bones need to be rigid levers for efficient locomotion, while the skull provides protection for the vulnerable brain. Structure of long bones in the limbs is regulated by the loads they experience during physiological activity (6), while the skull must retain mass and strength to resist damaging events despite the absence of any significant or regular loading. We have shown that in a human subject, the peak strains experienced in the skull even under extreme physiological circumstances do not reach 1/10th of the magnitude or rate those in the tibia of the same person (7). Such low strains would be perceived as disuse in a long bone, yet skull bone integrity is retained even in wasting conditions that deplete material in other parts of the skeleton. As all the bones in the skeleton experience the same circulating milieu of hormones and nutrients, it seems therefore that site-specific differences in regulation of bone formation and resorption must exist to account for regional differences. It might be considered that any differences between the skull and long bones are related to the embryological origins of the bone at the two sites, rather than their functional differences. This is unlikely because the clavicle is an example of a bone that is formed partly through endochondral ossification (like the long bones) and partly through intramembranous ossification (like the skull). The clavicles are bones which are known to adapt to function and lose bone mass through disuse, like long bones and unlike the skull (8), suggesting that intramembranous ossification is not invariably associated with a lack of sensitivity to disuse. Furthermore, the lateral end of the clavicle, which is the part formed by intramembranous ossification is affected by a bone wasting condition that has no effect on bone mass in the skull (9).

In order to identify the biological basis for site specific regulation of bone mass and structure we have used RNA sequencing to compare the transcriptomes of samples of skull and tibial bones from three species: mouse, rat, and rhesus macaque. Others have tried to address this question in the past, using bone samples (that included bone marrow) from young rats (10, 11) and mice (12), identifying several 100 genes that appeared regulated differently between sites. The cross-species design allowed us to focus our results on what we believe are broad biological mechanisms, and resulted in only a relatively small number of differences between sites that were shared across species and are therefore likely to reflect mechanisms in humans. The method we designed was to focus attention on enriched osteocytes populations, because osteocytes are widely accepted to be the mechanosensors in bone (13), responding rapidly to mechanical loading (14), and so influencing bone formation and resorption. More importantly, evidence has suggested that osteocytes, constituting more than 90% of bone cells, use the Wnt signaling pathway involving sclerostin and Dkk1, and crosstalk with the prostaglandin pathway in response to mechanical loading (15–17). Our current study has provided further evidence to support this notion but from a site-specific differences perspective.

## Materials and Methods

### Experimental Animals

Four-month old female C57BL/6 mice (Charles River, Harlow, UK), 6-month old female Wistar rats (Charles River, Harlow, UK), and 4–6 year old female rhesus macaques (*Macaca mulatta*) were used in this study. These ages represent bones which have undergone their periods of maximum growth rate for each species, and so transcriptome analysis should not be confounded by rapid growth of the skeleton (18). Long bones (tibias) and calvariae were dissected free of soft tissue after animals were euthanased. All procedures complied with the United Kingdom Animals (Scientific Procedures) Act 1986 (PPL 40/3499).

### Bone Tissue Preparation

To obtain highly enriched populations of osteocytes, tibias of mouse and rat were cut to expose the metaphysis and centrifuged briefly to remove bone marrow (1,500 g, 30 s). Calvarial samples were obtained from the rodents with scissors, removing the sutures and avoiding any marrow spaces (diploe). Samples were obtained from the Macaque bones with a hacksaw irrigated with normal saline. All samples were dissected free of periosteum and accessible surfaces were scraped with a scalpel. They were then immersed briefly in a 1 mg/ml solution of collagenase (Sigma Aldrich) for 3–5 min at 37°C to remove any adherent surface cells. Samples were then washed in saline before being snap frozen by complete submersion in liquid nitrogen and then stored at −80°C. Bone samples were pulverized using a Mikro-Dismembrator-S (Braun Biotech International GmbH Melsungen, Germany), in which the bone is shaken in a robust PTFE mill chamber with an 8 mm tungsten carbide ball (both cooled in liquid nitrogen before use, and again after adding the bone pieces, before agitation). The mill with tissue sample was placed within the Mikro-Dismembrator-S and set to shake at 2,500 rpm for 45 s. The weight of the fine bone powder was recorded and it was stored for RNA extraction.

### RNA Extraction From Bone

One milliliter of Trizol Reagent (Ambion) was added per 125 mg of pulverized tissue (typical tissue amounts 350–500 mg); samples were incubated in Trizol reagent for 10 min at room temperature. Samples were centrifuged at 500 g for 5 min at 4°C, the supernatant was removed carefully to ensure no debris was transferred. 0.3 ml of chloroform was added to each 1 ml of Trizol reagent, the sample was thoroughly mixed and allowed to incubate at room temperature for 5 min. The samples were centrifuged at 12,000 g for 20 min at 4°C. The colorless upper phase was collected and transferred to a separate tube, to which an equal volume of 70% ethanol was added and incubated at room temperature for 10 min.

The samples were then applied to spin cartridges from TRIzol Plus RNA purification kit (Ambion) following the manufactures instructions using the optional On-Column PureLink DNase (Invitrogen) treatment step. Samples were quantified using spectrophotometry (NanoDrop Thermo) and quality measured using a Bioanalyzer (Agilent). Only RNA with a RNA integrity number (RIN) above 8 was stored in −80°C and used for RNA-Seq. Samples used for analysis were one long bone and skull pair pooled from two rats, two long bone, and skull pairs each pooled from 3 mice, and 3 long bone, and skull pairs each from individual macaques.

### Library Preparation and Sequencing

cDNA library preparation and sequencing were performed by Eurofins Genomic (Ebersberg, Germany). From the total RNA sample, poly(A)+ RNA was enriched and randomized primer was used for first strand cDNA synthesis. Sequencing was performed in the 1 × 125 bp run mode, 6 samples/channel on Illumina HiSeq 2500 and generated 30 million reads/sample on average. The %Q30 of each samples were all above 86% and detailed quality control metrics were shown in Supplementary Table 1. Complete RNA sequencing data was submitted to the Gene Expression Omnibus under accession record GSE151971.

### RNA-Seq Data Processing and Functional Gene Annotation

RNA-seq data analysis pre-processing and functional gene annotation was performed by Bioinformatics Consultants (Stockholm, Sweden). Reference genome sequences and gene annotations for Rattus norvegicus (rn6) and Mus musculus (mm10) were downloaded from UCSC. For Macaca mulatta we used the MacaM genome assembly (https://www.unmc.edu/rhesusgenechip/index.htm) and its corresponding gene annotations (19). Exons of genes with multiple isoforms were merged according to their genomic coordinates using a custom Perl script. HISAT2 (v. 2.0.5) with default settings was used for mapping the sequencing reads to each respective genome (20). Sequencing reads were then counted on gene models with the HTSeq-count program (the –s flag set to “no”) (21). Differential gene expression was analyzed with the R package edgeR v. 3.16.5 for each species/individual separately (22). Any gene with zero read counts in at least one sample was removed, in order to lower the chance of quantifying expression from genes with incomplete annotation in one or more genomes. The trimmed mean of M-values (TMM) normalization was applied to raw read counts prior to testing for differential expression (23). Finally, differential expression was defined as genes with a false discovery rate <20% and an absolute fold change higher than 1.1 (24, 25). In the heat-map plots, raw read counts were TMM normalized, and a variance stabilizing transformation was applied (26). Heat-maps were rendered with the heatmap.2 function in the gplots R package. Three-way ortholog pairs across the three genomes were determined through their gene symbols with the same direction of gene expression. For functional gene annotation analysis, gene Ontology enrichment analysis was conducted on all identified three-way ortholog pairs with GOrilla (Gene Ontology enRIchment anaLysis and visuaLizAtion tool) (27), PANTHER (protein annotation through evolutionary relationship) classification system (28), and DAVID (Database for Annotation, Visualization and Integrated Discovery) (29). Gene Set Enrichment Analysis (GSEA) (30) was also conducted with GSEA v.2.2.3, ran with MSigDB v. 5.2 (gene set definitions) (31).

## Results

### Pipeline Design

**Figure 1:**
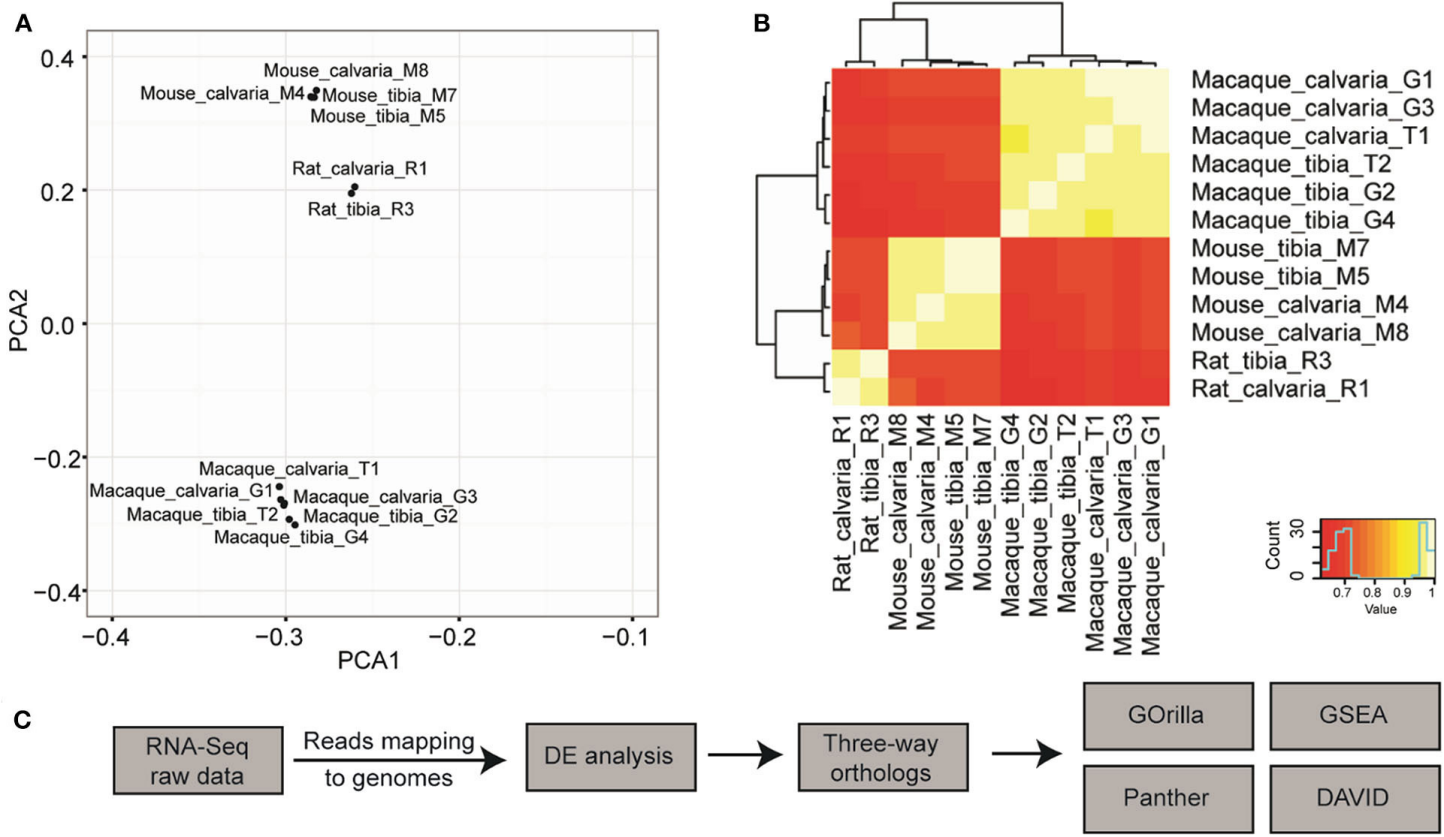
Pipeline design to discover cross species transcriptome signature of osteocytes in tibia and calvaria using RNA-Seq. **(A)** A principal component analysis (PCA) suggested that samples cluster according to species but not to tissue sites (data are based on ortholog triplets determined with OrthoDB). **(B)** Pairwise comparisons in a heat-map suggested samples from the same species but different sites have much more similar expression profile than samples of the same sites from a different species. **(C)** The pipeline to determine cross-species transcriptome signature: Raw RNA-seq data was aligned to relevant genomes using HISAT2. Differential expression (DE) was analyzed with the R package edgeR v. 3.16.5. Three-way ortholog pairs were determined based on gene symbols and the same direction of expression. Gene Ontology (GO) enrichment analysis was conducted with GOrilla, Panther classification system, and DAVID, in addition to Gene Set Enrichment Analysis with GSEA. After raw RNA-Seq data was mapped to relevant genomes, DE expression was performed for each species separately.

Orthologs across the three genomes were initially determined using OrthoDB (32). A principal component analysis (PCA) was conducted on the 12 samples using normalized expression of 1:1:1 orthologs. The result of this analysis showed that samples generally clustered according to species and not by sample site (Figure 1A), which indicates that the phylogenetic divergence is stronger than site differences. Similar results can also be seen through pairwise comparisons in a heat-map where samples from the same species but different sites have more similar expression profile than samples of the same sites from a different species (Figure 1B). This could be a consequence of analysis based on the limited 14,308 three-way orthologs that can be mapped 1:1:1 between Rat-Mouse-Macaque, due to incomplete genome annotations at the time OrthoDB was compiled. Therefore, to achieve enough power to detect transcriptome differences between different sites of skeleton across species, the differential expression (DE) analysis was carried out for each species/individual separately and orthologs were then determined using a simpler approach where the ortholog relationship is found through their gene symbols and same direction of gene expression. To this end, we propose a pipeline to determine transcriptome differences across species, which comprises of sequencing reads mapped using HISAT2, DE analysis with the R package edgeR v. 3.16.5, and gene symbols based three-way ortholog pairs determination, followed by Gene Ontology (GO) enrichment analysis conducted with GOrilla, Panther classification system, and DAVID, and Gene Set Enrichment Analysis with GSEA v.2.2.3 (Figure 1C).

### Differential Expression (DE) Analysis

**Figure 2:**
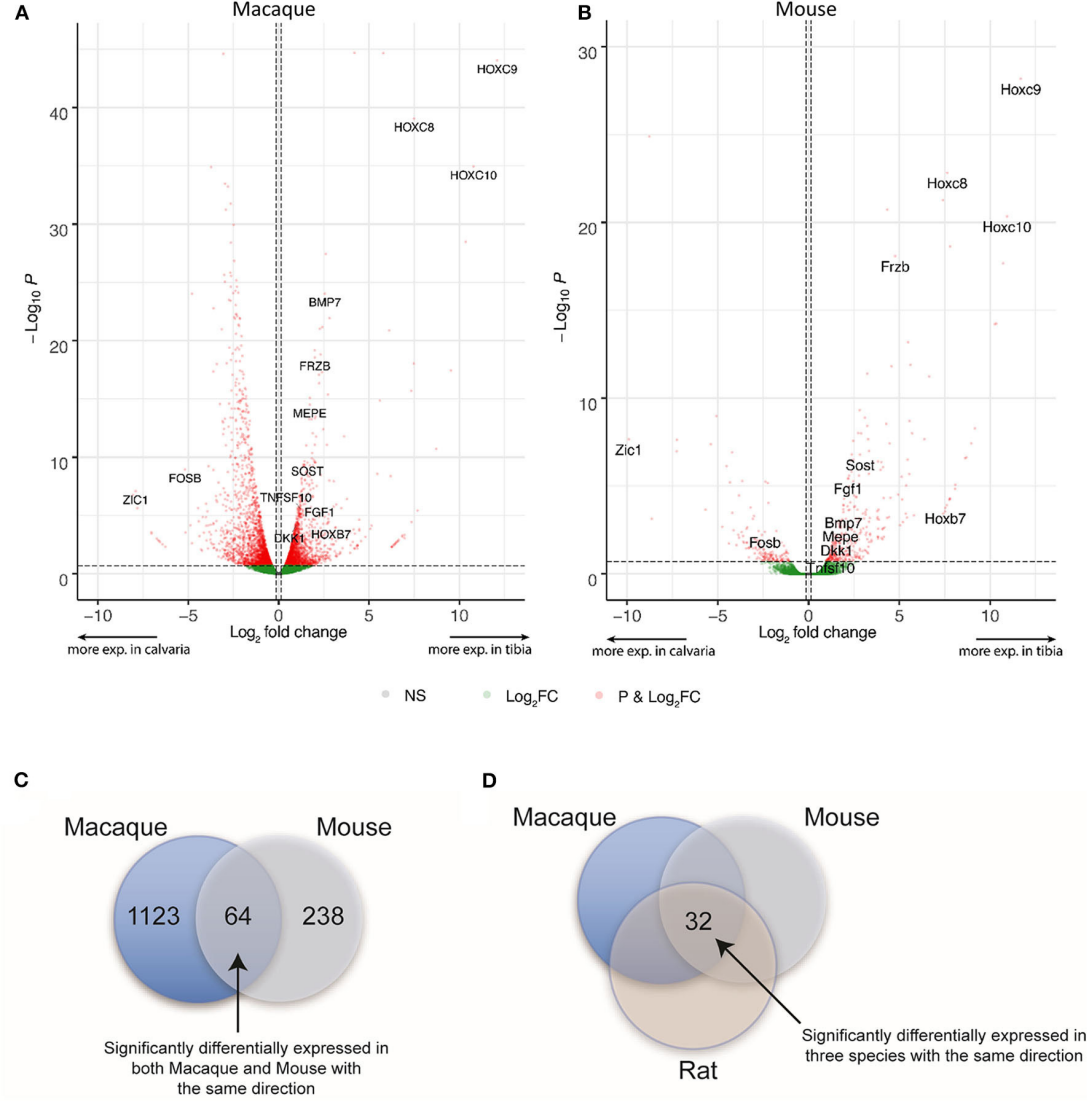
Differential expression (DE) and orthologs analysis. After raw RNA-Seq data was mapped to relevant genomes, DE expression was performed for each species separately. For macaque and mouse, genes with a false discovery rate <20% and an absolute fold change higher than 1.1 between samples from tibias and calvariae were defined as having significantly differential expression. **(A,B)** The distribution of transcriptomes were shown in the volcano plots built based on Log2 FC and FDR, with significant altered genes marked in red and specifically interested genes annotated. **(C)** There were 1,187 and 302 significantly DE genes in macaque and mouse, respectively. There are 64 genes sharing the same direction of expression in both macaque and mouse. **(D)** For rat, log2([cpm calvaria]/[cpm tibia]) > 2 was regarded as significantly DE, which produced 946 DE genes in rat. By comparing gene symbols, there were 32 genes common to all three species that shared same direction of gene expression.

DE expression was analyzed with the R package edgeR v. 3.16.5 for each species separately and differentially expressed genes were defined as genes with a false discovery rate <20% and an absolute fold change higher than 1.1 in mouse and macaque. This is to privilege sensitivity over specificity and highlight broad trends firstly (24). Therefore, 1,187 and 302 differentially expressed genes between tibias and calvariae were found in macaque and mouse, respectively (Figures 2A,B). By comparing gene symbols, there were 64 genes differentially expressed in both macaque and mouse with the same direction of change (Figure 2C). A conventional DE analysis with edgeR was not applied to the pair of rat samples as count dispersion could not be estimated. Instead, log2([cpm calvaria]/[cpm tibia]) >2 was selected as a threshold, which gave 946 DE genes in rat. By comparing gene symbols, there were 32 genes across all three species sharing same direction of gene expression (Figure 2D), with specifically interested genes annotated in Figures 2A,B. The list of genes with all quantitative data were ranked in a descending order in Table 1, based on fold changes of gene expression between calvariae and tibias.

**Table 1:** Genes sharing same direction of expression across three species (fold changes: calvaria over tibia).

	**Mouse**				**Macaque**				**Rat**		
	**logFC**	**logCPM**	**PValue**	**FDR**	**logFC**	**logCPM**	**PValue**	**FDR**	**CalvariaCPM**	**TibiaCPM**	**logFC**
HOXC9	−11.461493	4.0162301	8.01E-35	3.49E-31	−12.093574	5.0752142	7.15E-92	9.34E-88	0.1155338	27.7679897	−7.908963
IRX6	−2.279972	1.4212221	0.00174	0.1	−1.402094	2.4464369	0.00274	0.026	0.1155338	13.1126618	−6.826501
HOXC10	−10.738161	3.3064527	1.35E-26	2.93E-23	−10.803944	3.8065122	8.99E-47	1.96E-43	0.1155338	11.5699957	−6.645929
SEMA3E	−4.317833	3.5107122	2.17E-09	0.000000979	−1.256249	3.673995	0.000165	0.00273	0.1155338	7.9704415	−6.108272
HOXB7	−7.320998	0.1523548	0.0000104	0.00158	−3.350367	2.6031017	0.000000242	0.00000997	0.1155338	6.6848864	−5.854516
HOXC8	−8.578174	3.5611009	1.84E-27	4.81E-24	−7.493305	4.434895	3.35E-60	8.76E-57	0.1155338	5.9135534	−5.677638
TPBG	−2.449335	5.0845007	4.99E-13	3.83E-10	−1.781059	7.5889234	2.53E-13	3.93E-11	12.5931881	420.3765108	−5.060967
MAB21L2	−2.804119	1.6192526	0.000818	0.0595	−1.492797	4.7572048	1.11E-08	0.000000654	0.4621353	15.2981054	−5.048894
TBX18	−3.963703	2.9364331	8.65E-12	5.38E-09	−2.096215	0.7654256	0.0096	0.0676	0.693203	19.6689927	−4.826501
HOXC4	−5.411878	2.2721266	2.19E-10	0.000000124	−10.099025	3.1225023	1.19E-32	1.72E-29	0.3466015	8.356108	−4.591481
BMP7	−2.1987	4.5219894	9.17E-08	0.0000309	−2.434476	5.5399996	1.05E-13	1.77E-11	0.5776692	11.6985512	−4.339943
CNR1	−2.11486	1.6430663	0.000758	0.0563	−4.091067	0.6839788	0.0000267	0.000591	7.8563009	156.7091641	−4.318096
ISM1	−1.955616	5.9237582	9.81E-08	0.0000313	−1.340313	6.5592803	1.73E-11	1.92E-09	0.3466015	6.4277754	−4.21297
BNIP3	−2.108116	5.7664539	2.86E-08	0.0000116	−1.293536	6.6212956	0.0000138	0.000333	4.8524211	81.3756365	−4.06782
FGF1	−2.150014	5.0883888	4.88E-10	0.000000255	−1.229602	5.3376589	0.000000446	0.0000172	0.1155338	1.9283326	−4.060967
MEPE	−1.718209	9.4859049	0.0000159	0.00225	−1.415031	13.2160688	0.00000899	0.000231	395.2412537	5455.767199	−3.786977
DKK1	−1.675265	7.2375087	0.0000542	0.00673	−1.164476	7.5887557	0.0000137	0.000331	0.8087369	9.3845521	−3.536546
HOXB5	−4.060927	−0.4653591	0.00303	0.144	−3.905861	0.5559288	0.00205	0.021	0.1155338	1.2855551	−3.476004
RGS5	−1.14423	7.098667	0.000229	0.0213	−1.689813	9.7182745	1.62E-11	1.81E-09	1.0398045	9.1274411	−3.133898
EPS8L2	−1.885136	2.9125401	0.0000969	0.0103	−2.152398	0.3353361	0.0063	0.0494	1.9640752	13.3697728	−2.767053
MAPT	−1.752134	4.6115873	0.00112	0.0732	−1.352051	4.3814725	0.0000594	0.00117	13.0553235	86.5178568	−2.72836
SOST	−2.816051	12.2756927	7.77E-08	0.0000282	−1.592744	14.2838757	2.27E-12	2.9E-10	0.3466015	1.9283326	−2.476004
DOK5	−1.482686	2.8671925	0.0033	0.153	−1.322071	2.0727781	0.00451	0.0386	7.3941655	38.6952079	−2.387696
TNFSF10	−1.247027	3.5784116	0.00218	0.116	−1.149214	5.9821759	5.53E-08	0.00000268	0.8087369	3.9852207	−2.300917
VLDLR	−1.540378	6.2706147	0.00325	0.152	−1.199284	3.2402898	0.00158	0.0172	5.661158	27.7679897	−2.294254
SPARCL1	−1.409078	5.4723974	0.0000207	0.0029	−1.148213	6.921351	0.0000776	0.00146	109.1794752	517.4359193	−2.244679
HOXC5	−7.603457	0.3874172	0.00000282	0.000518	−6.992112	0.3315997	0.00000136	0.0000455	0.3466015	1.5426661	−2.154076
STC2	−1.726491	4.0178768	0.0000843	0.00922	−1.364338	2.3536737	0.00431	0.0372	2.5417444	11.1843292	−2.137588
NRN1	−3.169334	4.2319677	0.000000107	0.0000331	−1.856955	5.2077827	1.52E-08	0.000000857	1.9640752	8.613219	−2.132702
FRZB	−4.718328	5.175902	4.95E-22	6.47E-19	−1.280146	7.8660153	0.000431	0.00603	0.693203	2.9567767	−2.092675
FOSB	2.561736	2.5506365	0.00000287	0.00052	5.180265	6.7767497	1.01E-11	1.18E-09	13.7485265	1.9283326	2.833851
ZIC1	7.040348	2.6521869	9.23E-08	0.0000309	7.91982	1.0259272	1.09E-09	8.36E-08	1.1553384	0.1285555	3.167852

### Gene Ontology (GO) Enrichment Analysis

**Figure 3:**
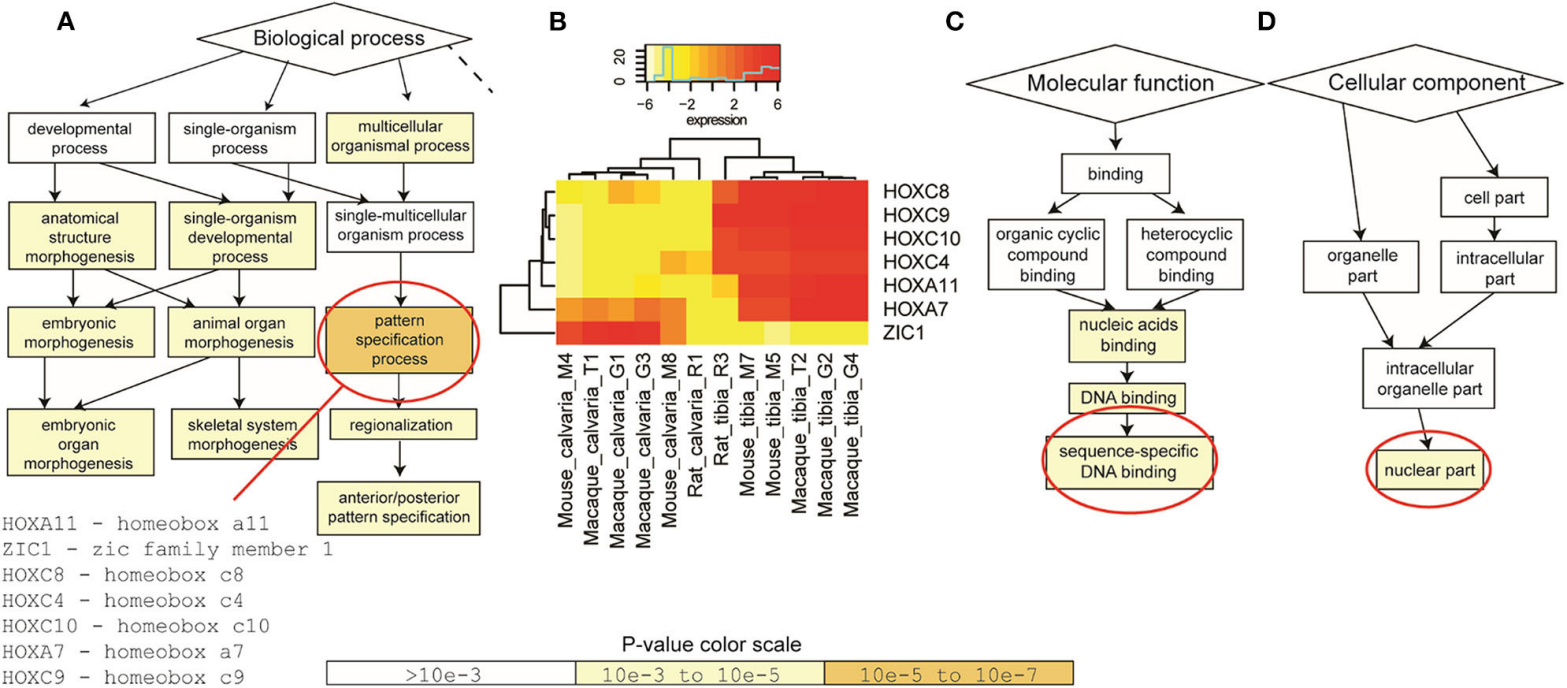
Gene Ontology (GO) enrichment analysis. GOrilla application was firstly performed on the 64 DE genes shared between macaque and mouse. DE genes in rat were then taken into account when quantitative analysis was performed. **(A)** At the level of biological process, a group of genes (HOXA7, HOXA11, HOXC4, HOXC8, HOXC9, HOXC10, ZIC1) were enriched in pattern specification process. **(B)** These genes were significantly up-regulated in tibias in all three species, apart from ZIC1. **(C)** These enriched DE genes mainly bind sequence-specific DNA as their molecular function and **(D)** located in nuclear part of the cellular component. *P*-value color scale for **(A,C,D)**.

To identify enriched GO terms in this list of shared DE genes, GO enrichment analysis using the web-based GOrilla application was performed first based on the 64 DE genes shared between macaque and mouse. DE genes in rat were then taken into account for quantitative analysis. Results suggested that a group of genes (HOXA7, HOXA11, HOXC4, HOXC8, HOXC9, HOXC10, ZIC1) are enriched in pattern specification process at the level of biological process (Figures 3A,B). These enriched genes are mainly binding sequence-specific DNA as their molecular function (Figure 3C) and located in nuclear part of the cellular component (Figure 3D).

**Figure 4:**
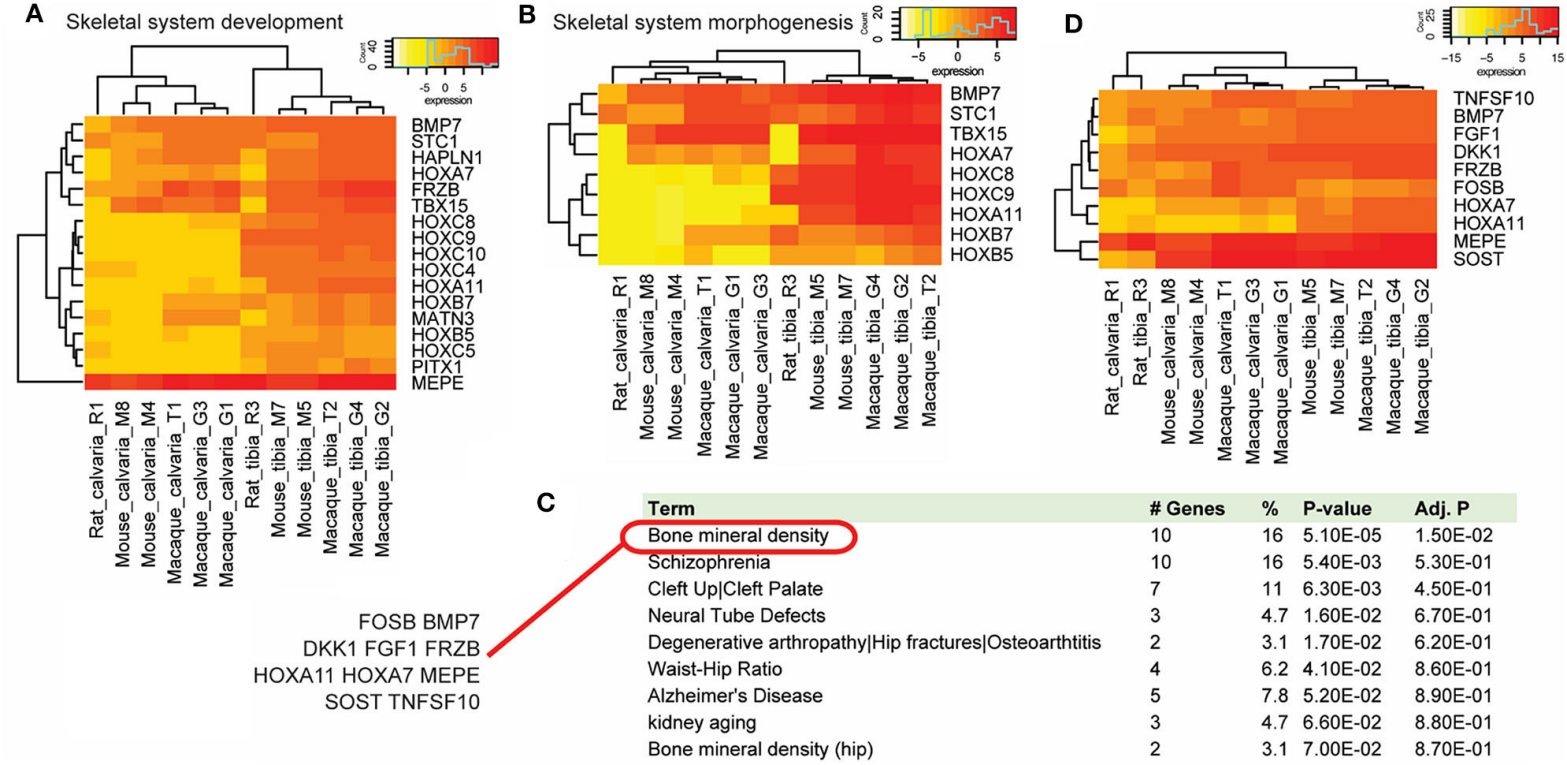
Gene function analysis. Gene function analysis with the PANTHER classification system and DAVID. PANTHER analysis suggested a list of DE genes that were up-regulated in osteocytes from tibias across species in biological processes of skeletal system development **(A)** and morphogenesis **(B)**. **(C)** Functional gene annotations using DAVID suggested that 10 cross-species DE genes (FOSB, BMP7, DKK1, FGF1, FRZB, HOXA11, HOXA7, MEPE, SOST, and TNFSF10) were implicated genes in bone mineral density related diseases, which ranks the top of the list. **(D)** Among these DE genes, only FOSB was down-regulated in tibias in all three species.

Gene function analysis with the PANTHER classification system suggested these DE genes mainly function in the biological processes of organismal development and morphogenesis (Table 2), not surprisingly in skeletal system development and morphogenesis (Figures 4A,B). This result is also consistent with the GSEA analysis which suggests in the tibia that there are multiple gene sets involved in skeletal development and morphogenesis. PANTHER protein class analysis confirms the results by GOrilla analysis that many of these genes belong to the family of homeobox transcription factor and are DNA binding proteins.

**Table 2:** Gene function in biological process analysis with the PANTHER classification.

**Term**	**Genes**
Regulation of animal organ formation	FGF1, DKK1, BMP7, HOXA11
Regulation of multicellular organismal development	HOXA7, MAPT, CNR1, FRZB, TBX18, SOST, FGF1, MEIS2, DKK1, HOXB7, BMP7, SEMA3E, VLDLR, GAL, MEPE, EAF2, HOXA11, TPBG
Regulation of multicellular organismal process	HOXA7, STC2, MAPT, CNR1, STC1, FRZB, TBX18, SOST, FGF1, MEIS2, DKK1, HOXB7, CLU, BMP7, SEMA3E, VLDLR, GAL, MFAP4, MEPE, EAF2, HOXA11, MYL4, TPBG
Embryonic skeletal system morphogenesis	HOXA7, TBX15, HOXC9, HOXB7, BMP7, HOXB5, HOXA11
Skeletal system morphogenesis	HOXA7, TBX15, STC1, HOXC9, HOXB7, BMP7, HOXB5, HOXC8, HOXA11
Animal organ morphogenesis	HOXA7, TBX15, STC1, FRZB, HOXC9, FGF1, HOXB7, BMP7, HOXB5, ZIC1, HOXC4, HOXC8, HOXA11
Single-organism developmental process	HOXA7, STC2, MAPT, TBX15, CNR1, STC1, FRZB, HOXC9, NRN1, HOXC10, TBX18, FGF1, MEIS2, DKK1, HOXB7, CLU, BMP7, CRABP1, MECOM, TNFSF10, SEMA3E, DOK5, VLDLR, HOXB5, GAL, AK4, CECR2, ZIC1, HOXC4, BNIP3, HOXC5, MEPE, MAB21L2, MATN3, HAPLN1, HOXC8, HOXA11, PITX1, ZIC2
Developmental process	HOXA7, STC2, MAPT, TBX15, CNR1, STC1, FRZB, HOXC9, NRN1, HOXC10, TBX18, FGF1, MEIS2, DKK1, HOXB7, CLU, BMP7, CRABP1, MECOM, TNFSF10, SEMA3E, DOK5, VLDLR, HOXB5, GAL, AK4, CECR2, ZIC1, HOXC4, BNIP3, SPARCL1, HOXC5, MEPE, MAB21L2, MATN3, HAPLN1, HOXC8, HOXA11, PITX1, ZIC2
Animal organ development	HOXA7, STC2, TBX15, STC1, FRZB, HOXC9, HOXC10, FGF1, MEIS2, DKK1, HOXB7, BMP7, MECOM, TNFSF10, VLDLR, HOXB5, AK4, CECR2, ZIC1, HOXC4, BNIP3, MEPE, MAB21L2, HOXC8, HOXA11, PITX1, ZIC2
Anatomical structure development	HOXA7, STC2, MAPT, TBX15, CNR1, STC1, FRZB, HOXC9, NRN1, HOXC10, TBX18, FGF1, MEIS2, DKK1, HOXB7, CLU, BMP7, CRABP1, MECOM, TNFSF10, SEMA3E, DOK5, VLDLR, HOXB5, GAL, AK4, CECR2, ZIC1, HOXC4, BNIP3, SPARCL1, HOXC5, MEPE, MAB21L2, MATN3, HAPLN1, HOXC8, HOXA11, PITX1, ZIC2
System development	HOXA7, STC2, MAPT, TBX15, CNR1, STC1, FRZB, HOXC9, NRN1, HOXC10, TBX18, FGF1, MEIS2, DKK1, HOXB7, CLU, BMP7, MECOM, TNFSF10, SEMA3E, DOK5, VLDLR, HOXB5, GAL, AK4, CECR2, ZIC1, HOXC4, BNIP3, HOXC5, MEPE, MAB21L2, MATN3, HAPLN1, HOXC8, HOXA11, PITX1, ZIC2
Multicellular organism development	HOXA7, STC2, MAPT, TBX15, CNR1, STC1, FRZB, HOXC9, NRN1, HOXC10, TBX18, FGF1, MEIS2, DKK1, HOXB7, CLU, BMP7, CRABP1, MECOM, TNFSF10, SEMA3E, DOK5, VLDLR, HOXB5, GAL, AK4, CECR2, ZIC1, HOXC4, BNIP3, HOXC5, MEPE, MAB21L2, MATN3, HAPLN1, HOXC8, HOXA11, PITX1, ZIC2
Single-multicellular organism process	HOXA7, STC2, MAPT, TBX15, CNR1, STC1, FRZB, HOXC9, NRN1, HOXC10, TBX18, SOST, FGF1, MEIS2, DKK1, HOXB7, CLU, BMP7, CRABP1, MECOM, TNFSF10, SEMA3E, DOK5, VLDLR, HOXB5, GAL, AK4, CECR2, ZIC1, HOXC4, BNIP3, HOXC5, MEPE, MAB21L2, MATN3, HAPLN1, HOXC8, HOXA11, PITX1, ZIC2
Multicellular organismal process	HOXA7, STC2, MAPT, TBX15, CNR1, STC1, FRZB, HOXC9, NRN1, HOXC10, TBX18, SOST, FGF1, MEIS2, DKK1, HOXB7, CLU, BMP7, CRABP1, MECOM, TNFSF10, SEMA3E, DOK5, VLDLR, HOXB5, GAL, AK4, CECR2, ZIC1, HOXC4, BNIP3, HOXC5, MEPE, MAB21L2, MATN3, FOSB, HAPLN1, HOXC8, HOXA11, PITX1, MYL4, ZIC2
Anatomical structure morphogenesis	HOXA7, TBX15, STC1, FRZB, HOXC9, NRN1, HOXC10, FGF1, DKK1, HOXB7, CLU, BMP7, SEMA3E, DOK5, VLDLR, HOXB5, CECR2, ZIC1, HOXC4, MAB21L2, HOXC8, HOXA11, PITX1
Skeletal system development	HOXA7, TBX15, STC1, FRZB, HOXC9, HOXC10, HOXB7, BMP7, HOXB5, HOXC4, HOXC5, MEPE, MATN3, HAPLN1, HOXC8, HOXA11, PITX1
Embryonic skeletal system development	HOXA7, TBX15, HOXC9, HOXB7, BMP7, HOXB5, HOXC5, HOXA11
Embryo development	HOXA7, TBX15, FRZB, HOXC9, HOXC10, DKK1, HOXB7, BMP7, HOXB5, CECR2, ZIC1, HOXC4, HOXC5, MAB21L2, HOXA11, PITX1
Embryonic organ morphogenesis	HOXA7, TBX15, FRZB, HOXC9, HOXB7, BMP7, HOXB5, ZIC1, HOXC4, HOXA11
Embryonic morphogenesis	HOXA7, TBX15, FRZB, HOXC9, HOXC10, DKK1, HOXB7, BMP7, HOXB5, CECR2, ZIC1, HOXC4, MAB21L2, HOXA11, PITX1
Embryonic organ development	HOXA7, TBX15, FRZB, HOXC9, HOXB7, BMP7, HOXB5, ZIC1, HOXC4, HOXA11
Anterior/posterior pattern specification	HOXA7, HOXC9, HOXC10, DKK1, HOXB7, HOXB5, HOXC4, HOXC5, HOXC8, HOXA11
Regionalization	HOXA7, HOXC9, HOXC10, DKK1, HOXB7, HOXB5, HOXC4, HOXC5, HOXC8, HOXA11
Pattern specification process	HOXA7, STC1, HOXC9, HOXC10, DKK1, HOXB7, BMP7, HOXB5, ZIC1, HOXC4, HOXC5, HOXC8, HOXA11
Negative regulation of developmental process	HOXA7, STC2, MAPT, FRZB, TBX18, SOST, MEIS2, DKK1, BMP7, SEMA3E, GAL, BNIP3, MEPE, EAF2
Positive regulation of transcription, DNA-templated	HOXA7, HOXC10, SOST, FGF1, MEIS2, HOXB7, BMP7, MECOM, HOXB5, GAL, ZIC1, EAF2, FOSB, HOXA11, PITX1, ZIC2
Positive regulation of nucleic acid-templated transcription	HOXA7, HOXC10, SOST, FGF1, MEIS2, HOXB7, BMP7, MECOM, HOXB5, GAL, ZIC1, EAF2, FOSB, HOXA11, PITX1, ZIC2
Positive regulation of RNA biosynthetic process	HOXA7, HOXC10, SOST, FGF1, MEIS2, HOXB7, BMP7, MECOM, HOXB5, GAL, ZIC1, EAF2, FOSB, HOXA11, PITX1, ZIC2
Regulation of transcription from RNA polymerase II promoter	HOXA7, TBX15, HOXC10, TBX18, SOST, FGF1, MEIS2, DKK1, HOXB7, BMP7, VLDLR, HOXB5, GAL, ZIC1, HOXC5, EAF2, FOSB, HOXC8, PITX1

Using the DAVID bioinformatics resources, functional gene annotations suggest that many of these DE genes are disease implicated genes. Bone mineral density ranks the top of the disease list and includes 10 shared DE genes (FOSB, BMP7, DKK1, FGF1, FRZB, HOXA11, HOXA7, MEPE, SOST, and TNFSF10) (Figure 4C), many of which associate with the Wnt signaling pathway. Among these DE genes, all but FOSB were up-regulated in tibias in all three species (Figure 4D).

## Discussion

The results of our experiments show a clearly defined set of genes that are regulated differently in the calvaria and tibia across mouse, rat, and macaque. Given the different physiological functions and responses to stimuli of bone cells at these two sites, there is a compelling case that these 32 candidates account for the bulk of this site specificity, although the mechanisms by which they do this are not completely clear. The results of the comparisons of the macaque paired samples are likely to be closer to human physiology than rodents because both are primates and therefore more related. However, the analysis of differences across species allows us to identify candidates that have a high likelihood of being broadly based regulators of mammalian site-specific differences rather than those only in any one of the separate species. Specifically, our analysis shows that samples cluster according to species more than to bone sample site (Figure 1A). We can interpret this to mean that the impact of phylogenetic divergence on these samples is stronger than any site-specific differences. This difference could be due to fundamental regulatory differences in the skeleton of different species, e.g., different functional units within compact bone. However, because of species differences, we believe the genes we have identified are representative of site specific rather than other differences. Using a single species for analysis could therefore affect the power to detect important general site-specific differences in expression, as any calvaria-tibia difference would have to be larger than the gene expression divergence between species. That inference is perhaps unsurprising, but it has important implications on likely relevance of our data when compared with studies performed in only one species (10, 11).

The bioinformatic analysis of the data provided robust results within and across species. Even where a conventional differential expression (DE) analysis could not be applied to the pair of rat samples (because count dispersion could not be estimated), we were able to use a different method to assess significant DE, identifying 946 DE rat genes. The segregation of our data into patterning genes (HOXA7, HOXA11, HOXC4, HOXC8, HOXC9, HOXC10, ZIC1) and 8 others known to be related to the regulation of bone mass and architecture (FOSB, BMP7, DKK1, FGF1, FRZB, MEPE, SOST, and TNFSF10).

We show that some of these genes (i.e., HOXC8, HOXC9, HOXC10) are almost exclusively expressed in tibias, at levels >64-fold higher than in calvariae. This is consistent with other studies that suggested HOX genes are pivotal in patterning the axial and appendicular skeleton, particularly for embryonic long bone development (33–39). This is consistent with their participation in cartilage differentiation in the process of endochondral ossification which is the way that long bones grow, but is not involved in the intramembranous ossification that leads to the formation of the flat bones of the skull (39–45). The involvement of a large group of HOX transcription factors indicates that the site-specific difference in regulating response to mechanical loading by osteocytes is partly a genetically determined baseline structure since embryonic stage.

Using the DAVID bioinformatics resources to identify DE genes involved in diseases, we found bone mineral density diseases ranking the top of the list and include implicated genes: BMP7, DKK1, FGF1, FRZB, MEPE, SOST, FOSB, and TNFSF10 (or TRAIL). Most of these genes code for secreted proteins and are up-regulated in osteocytes from tibias except FOSB. This list includes not only anabolic factors that would be expected to increase bone density (BMP7, FGF1, FRZB, and FOSB) but also catabolic factors (DKK1, SOST, MEPE, TNFSF10) with roles in bone remodeling, skeletogenesis and patterning (46–52). More interestingly, many of these genes, including FRZB (53, 54), BMP (55), FGF (56), DKK1 (57, 58), SOST (59), and MEPE (60–62), have long been shown to regulate skeletal development in association or crosstalk with the canonical Wnt signaling (55, 63). The identification of these bone mineral density and Wnt signaling-related, soluble proteins suggests osteocytes at different sites response to mechanical loading differently via potential paracrine and/or endocrine agents. Upon response to strain, osteocytes regulate bone remodeling with precisely tuned balance between anabolic and catabolic effects, targeting both osteoblasts and osteoclasts. Intriguingly, Sost^−/−^ mice were shown to have resistance to mechanical unloading-induced bone loss and exhibited high bone mass (64), which underscores our results that Sost is less expressed in the skull (with its insensitivity to disuse) but is more expressed in the tibia which requires significant mechanical loading to maintain mass. Moreover, this is also reflected by pathological conditions caused by disrupting transcription of the SOST gene and subsequently Wnt signaling, such as sclerosteosis and the van Buchem syndrome (VBCH). Both these two closely related diseases feature generalized sclerosis, including the enlargement of the skull which endures lower physical mechanical strains (65–67). All these indicate that the canonical Wnt signaling pathway dictates skeletal site differences through regulating bone's adaptive response to mechanical loading (16, 68). This could be achieved by osteocytes through a Sost-dependent feedback mechanism of maintaining quiescent bone-lining cells which are the main source of osteoblasts in adulthood (69, 70). However, other possible pathways could not be overlooked, for example, zinc finger protein of cerebellum (Zic) family member transcription factor Zic1, one of only two down-regulated gene in tibias in our study. Although not annotated by DAVID as a bone mineral density disease implicated gene, ZIC1 has been suggested a site-specific expression pattern by osteocytes and to act as a link between mechanosensing and Wnt signaling (71). The lower expression of ZIC1 in tibias suggests a potential negative feedback pathway in osteocyte in response to mechanical stimuli.

We recognize that the lack of additional technical validation and low number of biological replicates impacts on the strength of the conclusions that can be drawn from the study and the potential targets we have identified need to be confirmed and explored using *in vitro* models and transgenic mice in future. We can conclude that a relatively small number of genes are differentially expressed between two skeletal sites in multiple species: results that are consistent with some but not all differences found by others exploring site-specific differences in the skeleton and our data cast new light onto possible mechanisms for those differences as well as potential future clinical benefits. The approach we have used appears to have advantages over microarray studies in a single species, allowing greater refinement of data to identify key regulators of the site-specific characteristics of the skeleton. This has been underscored by a very recent study using scRNA-Seq, suggesting that osteoblasts isolated from calvaria and cortical long bone in mice have similar transcriptomes (12), although the authors suggest that changes after isolation of the cells may have contributed to the lack of differences identified. To impact upon bone pathology, therapies to make long bones behave more like the flat bones of the skull in respect of their susceptibility to loss in response to aging, disuse, and hormonal or nutritional changes could provide powerful methods to maintain bone strength throughout life and provide a strong biological explanation for the promising development of sclerostin antibodies for the treatment of osteoporosis (72, 73).

## Data Availability Statement

The datasets presented in this study can be found in online repositories. The names of the repository/repositories and accession number(s) can be found in the article/Supplementary Material.

## Ethics Statement

The animal study was reviewed and approved by the local Research Ethics Committee of the University of Sheffield (Sheffield, UK). All the procedures were performed under a British Home Office project license (PPL 40/3499) and in compliance with the UK Animals (Scientific Procedures) Act 1986.

## Author Contributions

TS and GR: study design. NW and CN: study conduct and data collection. NW, NL, and TS: data analysis and interpretation. NW and TS: drafting manuscript. NW, CN, GR, and TS: approving final version of manuscript.

## Conflict of Interest

The authors declare that the research was conducted in the absence of any commercial or financial relationships that could be construed as a potential conflict of interest.
