# Supplementary material for: Cross-Species RNA-Seq Study Comparing Transcriptomes of Enriched Osteocyte Populations in the Tibia and Skull
Source: Front Endocrinol (Lausanne). 2020 Sep 24;11:581002. doi: 10.3389/fendo.2020.581002 (PMC7543096; doi:10.3389/fendo.2020.581002)
Supplement: Supplementary file 1 [file Table_1.docx]

Supplementary Table 1. Metrics of Sequencing Results

| Lane | Sample | Index | Yield (Mbp) | #Cluster | %Q30 | Mean Q |
| --- | --- | --- | --- | --- | --- | --- |
| 3 | M4 | ATCACG | 3 519 | 28 151 014 | 86.37 | 33.19 |
| 3 | M5 | ACTTGA | 3 865 | 30 918 272 | 88.43 | 33.97 |
| 3 | M7 | CGATGT | 3 334 | 26 672 680 | 88.31 | 33.89 |
| 3 | M8 | GATCAG | 3 230 | 25 836 835 | 88.81 | 34.03 |
| 3 | R1 | TGACCA | 3 541 | 28 328 809 | 88.28 | 33.93 |
| 3 | R3 | TAGCTT | 3 275 | 26 202 885 | 87.37 | 33.67 |
| 3 ∑ 20 764 ∑ 166 110 495 | | | | | | |
| 4 | G1 | TTAGGC | 2 905 | 23 236 452 | 90.27 | 34.40 |
| 4 | G2 | GATCAG | 3 401 | 27 204 378 | 90.23 | 34.34 |
| 4 | G3 | TGACCA | 3 032 | 24 255 735 | 88.72 | 33.85 |
| 4 | G4 | TAGCTT | 3 298 | 26 381 528 | 89.31 | 34.06 |
| 4 | T1 | CGATGT | 3 190 | 25 520 142 | 89.90 | 34.25 |
| 4 | T2 | ACTTGA | 3 661 | 29 290 443 | 90.39 | 34.44 |
| 4 ∑ 19 487 ∑ 155 888 678 | | | | | | |
| **∑ 40 251 ∑ 321 999 173** | | | | | | |

Remarks:

• Sequences are demultiplexed according to the 6 bp index code with 1 mismatch allowed.

• "Yield (Mbp)": number of bases called in mega bases.

• "%Q30": represents the percentage of bases with a quality score of at least 30 (inferred base call accuracy of 99.9%).
